# SIB Literature Services: RESTful customizable search engines in biomedical literature, enriched with automatically mapped biomedical concepts

**DOI:** 10.1093/nar/gkaa328

**Published:** 2020-05-07

**Authors:** Julien Gobeill, Déborah Caucheteur, Pierre-André Michel, Luc Mottin, Emilie Pasche, Patrick Ruch

**Affiliations:** SIB Text Mining group, Swiss Institute of Bioinformatics, 1206 Geneva, Switzerland; BiTeM group, Information Sciences, HES-SO / HEG Geneva, 1227 Carouge, Switzerland; BiTeM group, Information Sciences, HES-SO / HEG Geneva, 1227 Carouge, Switzerland; SIB Text Mining group, Swiss Institute of Bioinformatics, 1206 Geneva, Switzerland; BiTeM group, Information Sciences, HES-SO / HEG Geneva, 1227 Carouge, Switzerland; SIB Text Mining group, Swiss Institute of Bioinformatics, 1206 Geneva, Switzerland; BiTeM group, Information Sciences, HES-SO / HEG Geneva, 1227 Carouge, Switzerland; SIB Text Mining group, Swiss Institute of Bioinformatics, 1206 Geneva, Switzerland; BiTeM group, Information Sciences, HES-SO / HEG Geneva, 1227 Carouge, Switzerland

## Abstract

Thanks to recent efforts by the text mining community, biocurators have now access to plenty of good tools and Web interfaces for identifying and visualizing biomedical entities in literature. Yet, many of these systems start with a PubMed query, which is limited by strong Boolean constraints. Some semantic search engines exploit entities for Information Retrieval, and/or deliver relevance-based ranked results. Yet, they are not designed for supporting a specific curation workflow, and allow very limited control on the search process. The Swiss Institute of Bioinformatics Literature Services (SIBiLS) provide personalized Information Retrieval in the biological literature. Indeed, SIBiLS allow fully customizable search in semantically enriched contents, based on keywords and/or mapped biomedical entities from a growing set of standardized and legacy vocabularies. The services have been used and favourably evaluated to assist the curation of genes and gene products, by delivering customized literature triage engines to different curation teams. SIBiLS (https://candy.hesge.ch/SIBiLS) are freely accessible via REST APIs and are ready to empower any curation workflow, built on modern technologies scalable with big data: MongoDB and Elasticsearch. They cover MEDLINE and PubMed Central Open Access enriched by nearly 2 billion of mapped biomedical entities, and are daily updated.

## INTRODUCTION

It has been repeatedly stated in the last decade that biocurators need (semi-)automated support from text mining technologies for managing the growing amount of biomedical knowledge described in the scientific literature (1,2). Pointed issues include scalability and interoperability. Indeed, to populate structured databases, biocurators have to retrieve information in a growing amount of publications, and then to capture valuable knowledge by the identification and normalization of the involved biomedical entities (3). Between 2009 and 2019, the number of yearly published citations in MEDLINE has grown from 880 000 to 1 400 000, while the number of concepts describing gene functions in the Gene Ontology vocabulary has grown from 20 000 to almost 50 000. Many initiatives have attempted to promote interactions between the text mining and the biocuration communities, from local collaborations (4,5) to international efforts, such as the BioCreative challenges (6,7).

In 2020, biocurators have at their disposal plenty of good systems and ergonomic Web interfaces for identifying and visualizing biomedical entities in literature, both MEDLINE citations (8) and PMC full texts (9). Among the most popular are PubTator central (10), Textpresso central (11) and SciLite (12) which empowers Europe PMC (13). These systems enrich texts with biomedical entities, either found by lexical mapping from a reference vocabulary, or produced by state-of-the-art Named Entity Recognition tools, or exported from expert-curated databases. For instance, all occurrences of ‘hepatocellular cancer’ or ‘hepatic neoplasm’ in a text can be identified and normalized with the unique MeSH concept ‘D008113: Liver Neoplasms’. These annotations are then highlighted for readers in the dedicated search engine Web interface, and can often be used for complementing keywords in the search process for a better recall – as all synonyms will be mapped under a unique concept. Some systems allow the user to search in sub-collections, or to organize the results in clusters of annotated entities (14,15).

Yet, many systems start with a PubMed query, which has advantages but is limited by strong Boolean constraints (AND/OR operators), and returns results in anti-chronological order. Beyond query terms, some semantic search engines aim at delivering ranked results, based on relevance for the user's information need. Such systems typically exploit words weighting algorithms for favouring most informative keywords, such as the recent relevance sorts in PubMed (16) and EuropePMC (17). Among other approaches are learning-to-rank algorithms based on user feedback (18), on user clickthrough history (19), on learning data (20) or on journal's impact factor and authors’ contribution (21). Yet, such search engines are not designed for delivering relevant articles for a specific curation workflow, and allow very limited control on the search process, even in their advanced search mode. Literature triage is a major benefit for curation teams as, for example, for the curation of UniProt, 90% of MEDLINE is out of the scope, while a maximum of 2–3% is relevant (5). A couple of works recently focused on literature triage based on deep learning algorithms, based on convolutional neural networks (22,23), resulting on higher precisions in result sets.

We present the Swiss Institute of Bioinformatics Literature Services (SIBiLS), which aims at providing precision Information Retrieval in the biological literature. SIBiLS do not provide Web interfaces, but fully operational and free RESTful APIs. Within SIBiLS, a local mirror of MEDLINE and the PMC Open Access subset is maintained, and daily updated. All contents are parsed and semantically enriched with automated annotations of biomedical entities. Biomedical entities are identified by lexical mapping from a growing set of standardized and legacy vocabularies. In January 2020, almost 2 billion annotations produced with a dozen of vocabularies populate SIBiLS. Parsed contents and annotations for MEDLINE citations and PMC full texts are stored in a JATS BioC json format, and accessible via the fetch APIs. They are also indexed in Lucene Elasticsearch search engines. The search APIs allow to interrogate the search engines with fully customizable queries, exploiting the power of the rich Lucene query language (24). SIBiLS are ready to empower literature triage, and to be efficiently integrated in any curation workflow, built on modern technologies scalable with big data: MongoDB and Lucene Elasticsearch.

## SYSTEM DESCRIPTION

The overall architecture of SIBiLS is presented in the graphical abstract.

### The content collecting and parsing pipeline

Both collections (MEDLINE the PMC Open Access subset) are updated daily via the US National Library of Medicine (NLM) and National Center for Biotechnology Information (NCBI) FTP servers. The SIBiLS pipeline uses local XML parsers. Their main role is to product a simple json record, representing the different fields specific to the citation (such as abstract or MeSH terms) or the full text (such as hierarchical structure or figure captions). The json document representations are stored in a MongoDB database, ready to be accessed by the automatic annotation tool and the search engine. In particular, PMC full texts delivered by the NCBI are XML files complying with the Journal Article Tag Suite (JATS) DTD (https://jats.nlm.nih.gov/archiving/tag-library). Although the JATS DTD provides a finite number of tags to describe an article, there is still a huge number of ways to describe the same document. For the parser, the challenge consists in reducing the complexity of the JATS representation without losing textual content nor important structural aspects of the documents.

Some implementation aspects related to these goals are described hereafter. The parser handles tables and figures so that caption, footer notes but also table contents are parsed and made available to the next steps of the pipeline, because these textual contents are known to be a good mine for annotations (25). The most challenging aspect of the parsing is to deal with the complex structure of embedded XML tags. The JATS DTD is very permissive in terms of which tag can contain which other tags and allows recursion. For example, a section can contain paragraphs, figures, tables, and/or lists but also (sub) sections, and a paragraph can embed lists, figures and graphs as well. The SIBiLS parser turns the hierarchical structure of the document into a flat list of sections, each of which acting as a container for a flat list of multiple contents. The final representation is simple, easy to process and reflects the original sequential position of text elements as well as their hierarchical level in the document structure. Each content is tagged (paragraph, figure-footer, table-content, list-item, etc.) and its textual content constitutes the input for the annotation tool described below. The parsing component is freely accessible online (https://github.com/bitem-heg-geneve/jats-parser).

### The automatic annotation pipeline

For identifying biomedical entities in text, SIBiLS use a growing set of standardized and legacy biomedical vocabularies. In January 2020, a dozen of vocabularies are exploited, including Drugbank (26) for drugs, the NCI Thesaurus (27) for diseases, neXtProt (28) for human genes or the NCBI taxonomy (29) for species. The SIBiLS automatic annotations are produced with lexical mapping, improved by state-of-the-art text pre-processing. Synonyms are collected across vocabularies in order to improve the entities mapping.

The annotation pipeline is divided in four steps. (i) Extraction: for a given document, the parsed representation is loaded from the MongoDB database, and fields of interest are extracted. Some are common to both collections (title, abstract, keywords), while others are more specific like MeSH terms for MEDLINE citations, or elements relative to figures or table for full texts. (ii) Tokenization: each sentence is broken down into individual words and words n-grams (sequences of words). (iii) String pre-processing: it consists in dealing with special characters. For example, words containing a dash are transformed to a set of additional words (‘B-RAF’ becomes ‘B’, ‘RAF’, ‘BRAF’), and symbols are replaced by corresponding Latin alphabet letters (‘β’ becomes ‘b’). (iv) Annotations: they are produced thanks to lexical mapping between the pre-processed strings and the exploited vocabularies. For each vocabulary concept, the set of possible strings (e.g. preferred term, synonym) is tentatively matched in the text, and eventually results in annotations. For each annotation, different features are saved, such as the concept id, the matching sentence or the characters offsets. The automatic annotation process is applied daily to updated contents, and up to monthly to the full collections for frequently updated vocabularies (such as neXtProt). Finally, automatic annotations are built in json format, and stored into a MongoDB database, ready to be accessed by the search engines and the fetch API.

### Search engines and APIs design

The content parsing and automatic annotation pipelines deliver up-to-date json representations in a MongoDB database. Then, both representations are combined and indexed in two Lucene Elasticsearch search engines: one for MEDLINE citations, the other for PMC full texts. For computational efficiency and optimized response times, nested fields are flattened, with values separated by a vertical bar ‘|’. In particular, a field ‘annotations_str’ is built for gathering vocabulary identifiers for all automatic annotations mapped in the document, along with their type. For example, when the gene ‘ZBED1’ was mapped in the text thanks to the neXtProt vocabulary, the annotations_str field contain ‘gene NP_NX_O96006’. The Elasticsearch engines are daily updated.

A Tomcat Web server handles requests from APIs clients. For the content fetch APIs, parsed contents and their annotations are beforehand converted and stored in BioC format, allowing the API to return requested data in optimized response times. The maximum number of documents that can be requested per call is 1000. The content search APIs submit a Lucene query to the Elasticsearch engines, and return the engine result set in its native json format.

### USAGE

SIBiLS can be accessed via RESTful APIs, for fetching annotated contents or searching in annotated collections. Endpoints, parameters, and data formats are detailed in the services home page (http://candy.hesge.ch/SIBiLS/). Python scripts samples for calling the services and loading the response in variables are also provided.

#### Fetch APIs

They allow to retrieve annotated contents from MEDLINE or PMC Open Access. The input is a set of pmids, or pmcids (up to 1000 per request). The output is a set of parsed and annotated contents, in both JATS and BioC formats. For MEDLINE citations, delivered and annotated fields include for example abstracts, or MeSH terms; for PMC full texts, paragraphs provided with their hierarchical level in the document structure, or figure captions. Annotations are delivered with many features including the type of the mapped entity (drug, gene, disease…), the vocabulary used, the vocabulary unique identifier and preferred term, or the mapping characters offsets.

#### Search APIs

They allow to perform a fully customizable search for valuable documents in MEDLINE or PMC Open Access. The power of these services is based on the efficiency of Elasticsearch engines, and on the rich Lucene query language (https://www.elastic.co/guide/en/kibana/current/lucene-query.html), which allows to investigate a large panel of searching strategies. For example: basic search with keywords or entity identifiers (‘ZBED1’ or ‘NP_NX_O96006’), searches in specified fields (‘figures_captions: ZBED1’ or ‘tables: mapped treatments’), boosting fields or query parts, Boolean, fuzzy or wildcard queries (‘BRCA*’)… The input is thus a Lucene json query. The output is the Elasticsearch ranked result set in its native json format; for each document (up to 10 000 per request), a relevance score and the indexed content.

## USE CASES

### Case 1: literature triage for curation teams

The SIB Literature Services are currently integrated in various specific workflows from different curation teams. The services are delivering literature triage with success to the DisProt curation team (30). With regard to the neXtProt database, SIBiLS empower the local neXtA5 curation interface (31) for the SIB CALIPHO group. This group is interested in the curation of neXtProt genes for several curation axis: biological processes and molecular functions with the Gene Ontology, and diseases with the NCI thesaurus. The neXtA5 interface submits customized Lucene queries to SIBiLS, focusing on a gene name and the presence of curatable mapped entities, and proposes potentially relevant articles and entities. The CALIPHO curation team evaluated and reported on the use of this interface for delivering literature triage and curatable entities, handled by two curators (32). As presented in Table 1, 63–67% of the proposed articles were accepted by both curators, and only 17 to 20% were rejected by both. Furthermore, 22% of the proposed curatable concepts were accepted for direct curation in neXtProt for both axis (25–35% after small changes). Although 65–75% of proposed concepts were discarded for curation, many are actually not in the scope of the neXtProt curation; while being useful for the literature triage, they can be filtered by the interface at display time.

**Table 1.** Evaluation of SIBiLS for curation support of the neXtProt database by the SIB Calipho group

Identified articles			
Curation axis	Accepted by both	Accepted by one	Rejected
Biological processes	162 (**67%**)	39 (**16%**)	41 (**17%**)
Diseases	152 (**63%**)	48 (**17%**)	42 (**20%**)
Identified concepts			
	Accepted for curation	Modified for curation	Rejected for curation
Biological processes	699 (**22%**)	413 (**13%**)	2061 (**65%**)
Diseases	1094 (**22%**)	146 (**3%**)	3727 (**75%**)

### Case 2: information retrieval for precision medicine

The 2019 TREC Precision Medicine track focused on the case of providing clinical decision support for cancer patients with genetic variations that might impact the choice of treatment (http://www.trec-cds.org/). The test set consisted in 40 topics designed by precision oncologists. For each topic were provided a disease (e.g. for topic 1: ‘melanoma’), a gene (‘B-RAF’), a variant (‘E586K’), and demographic information about the patient (‘female, 64 yo’). The goal of the scientific abstracts sub-task was to identify, in MEDLINE, relevant articles for the treatment, prevention, and prognosis of the disease for the given patient. Our group participated and obtained competitive results among the top three (33).

For this article, we reproduced the evaluation in order to compare Information Retrieval with PubMed and with SIBiLS. Under the same conditions of competition, we interrogated PubMed and SIBiLS with, for each topic, a query containing the disease, the gene and the variant. PubMed was interrogated both with the standard chronological sort and the relevance sort; PubMed is not a strict Boolean search engine, as its query processing allows to identify MeSH terms (such as ‘D008545: melanoma’) and to use this normalization for better results. In parallel, the SIBiLS search API was interrogated with the query, and with an extended query where the diseases and genes were normalized with the NCI thesaurus and neXtProt, and searched in the annotations (e.g. ‘C3224: melanoma’ and ‘NX_P15056: braf’ in the annotations_str field). The evaluation was done thanks to the relevance judgements published after the competition (5,544 abstracts judged as relevant), and with standard IR metrics (34). Results are presented in Table 2. For R-Prec and MAP, two of the most discriminative metrics usually exploited by TREC, SIBiLS outperforms PubMed (relevance sort) by +67%. With SIBiLS, the number of relevant retrieved citations is +114% higher, and the Precision of top 20 returned citations is remarkably good (50%) for such a complex information need.

**Table 2.** Evaluation of PubMed and SIBiLS for Information Retrieval with the TREC 2019 Precision Medicine benchmark

Search engine	Relevant retrieved	P20	R100	R-Prec	MAP
PubMed	1437	0.23	0.18	0.14	0.10
PubMed (relevance sort)	1624	0.33	0.21	0.18	0.15
SIBiLS	3212	0.47	0.29	0.27	0.22
SIBiLS (normalized queries)	3468	0.50	0.31	0.30	0.25
**Improvement**	**+114%**	**+52%**	**+48%**	**+67%**	**+67%**

## CONCLUSION

We have described the SIB Literature Services, RESTful APIs for personalized Information Retrieval in fully annotated MEDLINE and PMC mirrors, indexed by Lucene search engines. Both collections are updated daily. The parsed citations and full texts are semantically enriched with entities lexically mapped in a growing set of legacy onto-terminological descriptors, resulting in a total of almost 2 billion annotations in January 2020. Parsed and annotated documents can be delivered in both JATS and BioC json format. The services have been used and evaluated to support the curation of genes and gene products, by delivering customized literature triage engines to different curation teams. Thanks to fully customizable searches, based on the rich Lucene query language, SIBiLS can exploit the density of annotations in order to propose an improved ranking function compared to the state of the art. The services are freely available, and scalable with big data, built on the modern technologies MongoDB and Elasticsearch. Finally, SIBiLS can be easily integrated into the local mining and curation pipelines of our remote users.

## DATA AVAILABILITY

The Swiss Institute of Bioinformatics Literature Services (SIBiLS) are publicly available at https://candy.hesge.ch/SIBiLS/.

## Notes

Present address: SIB Text Mining group, Swiss Institute of Bioinformatics, Rue Michel-Servet 1, 1206 Genève, Switzerland.
